# Modeling Dynamic Systems with Efficient Ensembles of Process-Based Models

**DOI:** 10.1371/journal.pone.0153507

**Published:** 2016-04-14

**Authors:** Nikola Simidjievski, Ljupčo Todorovski, Sašo Džeroski

**Affiliations:** 1 Department of Knowledge Technologies, Jožef Stefan Institute, Ljubljana, Slovenia; 2 Jožef Stefan International Postgraduate School, Ljubljana, Slovenia; 3 Faculty of Administration, University of Ljubljana, Ljubljana, Slovenia; Harbin Institute of Technology Shenzhen Graduate School, CHINA

## Abstract

Ensembles are a well established machine learning paradigm, leading to accurate and robust models, predominantly applied to predictive modeling tasks. Ensemble models comprise a finite set of diverse predictive models whose combined output is expected to yield an improved predictive performance as compared to an individual model. In this paper, we propose a new method for learning ensembles of process-based models of dynamic systems. The process-based modeling paradigm employs domain-specific knowledge to automatically learn models of dynamic systems from time-series observational data. Previous work has shown that ensembles based on sampling observational data (i.e., bagging and boosting), significantly improve predictive performance of process-based models. However, this improvement comes at the cost of a substantial increase of the computational time needed for learning. To address this problem, the paper proposes a method that aims at efficiently learning ensembles of process-based models, while maintaining their accurate long-term predictive performance. This is achieved by constructing ensembles with sampling domain-specific knowledge instead of sampling data. We apply the proposed method to and evaluate its performance on a set of problems of automated predictive modeling in three lake ecosystems using a library of process-based knowledge for modeling population dynamics. The experimental results identify the optimal design decisions regarding the learning algorithm. The results also show that the proposed ensembles yield significantly more accurate predictions of population dynamics as compared to individual process-based models. Finally, while their predictive performance is comparable to the one of ensembles obtained with the state-of-the-art methods of bagging and boosting, they are substantially more efficient.

## Introduction

Models are vital instruments for investigating how constitutive elements interact in the complex dynamic systems observed in nature. Scientists incorporate expert knowledge and statistical methods to recreate the observed behavior and find patterns in the measured data, which in turn results in a model of a dynamic system. The model can be simulated- both to describe and to predict its states under various conditions.

The process-based modeling paradigm, employed in this paper, follows the above principles by automatically modeling the behavior of dynamic systems using time-series measurements and domain-specific modeling knowledge. The resulting process-based models offer both an understandable formalism of the system’s structure and a mathematical formulation allowing for its simulation. The former provides a high-level representation of the modeled system in terms of entities and processes, i.e., the elements in the system and their interactions, respectively. The latter, transforms these entity/process components into a system of ordinary differential equations (ODEs). ODEs are a widely accepted modeling formalism. They allow for long-term simulation of dynamic systems, which requires a minimal input consisting the initial value of the system’s state and data corresponding to the system’s exogenous variables.

Often, establishing models that provide an accurate prediction of the behavior of a dynamic system, require an expert intervention, which is time consuming and expansive. In contrast, the process-based modeling paradigm allows for automated construction of such models [1–4]. While these automatically constructed models can be employed for long-term prediction, the respective studies mostly focus on constructing models that provide a detailed and precise description of the observed behavior. Unfortunately, the resulting models often fail to accurately predict the subsequent states of the system. In this paper, we focus on the task of improving the predictive performance of process-based models by constructing ensembles.

An ensemble model is a combination of predictive models, which is expected to lead to more accurate prediction than the one obtained with a individual model [5]. Ensembles are a standard approach in machine learning for improving the predictive performance of models. They are usually employed in the context of the learning tasks of classification and regression [6, 7] to address the problems of over-fitting, high dimensionality, or missing features in the training data. Recent studies by Simidjievski et al. [8, 9] have introduced ensembles in the context of process-based models. This has allowed more accurate long-term prediction of the behavior of dynamic systems. The ensembles yield a significant gain in predictive performance over the individual process-based models. However, this performance gain comes at the cost of increased computational complexity.

The main contribution of this paper is a novel efficient method for learning ensembles of process-based models, that still accurately predict long-term behavior of dynamic systems. The method learns ensembles by sampling the library of domain-knowledge in a manner similar to the standard ensemble learning method of random subspaces [10]. We conjecture that such ensembles will outperform individual process-based models in terms of their accuracy of long-term predicting the system’s behavior, and will overcome the computational limitations of the existing methods for learning ensembles of process-based models based on data sampling. To test the validity of this conjecture, we perform an extensive empirical evaluation of the implemented method on the task of modeling and predicting population dynamics in aquatic ecosystems. This empirical evaluation will allow us to identify the most appropriate design decisions within the algorithm for learning such ensembles of process-based models. It will also allow us to compare the predictive performance of the new method to the performance of the existing methods of bagging and boosting of process-based models.

The remainder of the paper is organized as follows. In the next section, we provide an overview of the related work from the areas of process-based modeling and ensemble learning. Section *Process-based models* introduces the process-based modeling paradigm and its latest implementation ProBMoT (Process-Based Modeling Tool), by illustrating its use on a simple modeling task from the domain of population dynamics. Section *Ensembles of process-based models* presents the extension of ProBMoT for learning ensembles of process-based models, focusing on learning ensembles by sampling the library of domain knowledge. Next, we present the experimental setup for evaluating the developed methods on three tasks of predictive modeling of population dynamics in aquatic ecosystems, i.e., the ecosystems of Lake Bled in Slovenia, Lake Kasumigaura in Japan and Lake Zurich in Switzerland. Section *Results* presents the findings of the empirical evaluation and gives a complexity analysis for learning ensembles of PBMs. In the next section, we discusses the results in the context of related research. Finally, the last section concludes the paper and suggests directions for further work.

### Related research

The work presented in this paper builds upon our previous research on ensembles of processes-based models [8, 9]. These studies tackle the challenge of learning ensembles by sampling the training data. However, while the empirical evaluations in the respective studies have shown that ensembles of process-based models lead to significant gains in predictive performance, the process of learning such ensemble models is very computationally demanding.

In contrast, here we introduce a novel approach which will learn ensembles by sampling the library of domain knowledge. The work presented in this study, is closely related to the work performed by Bridewell et al. [11], where the authors report on generating an ensemble of PBMs by fusing the structures of the individual constituents. The empirical evaluation of the respective study shows that such ensembles have improved performance in a descriptive setting, i.e., in explaining the observed behavior, whereas their ability for long-term prediction (i.e., outside the scope of the training data time intervals) are not reported.

In a broader sense, this paper extends the scope of the state-of-the-art in equation discovery [12, 13], related to automatically obtaining descriptive models from domain knowledge. Our work comes closer to the inductive process modeling paradigm [14, 15] which mainly tackles the problem of automatically obtaining explanatory models of a dynamic system in a process-based representation. This paradigm has proven to be successful for a variety of modeling tasks of population dynamics in a number of real-world domains for explaining the observed behavior of the modeled system [1–4]. However, focusing on the provision of detailed and accurate descriptions of the observed systems, the models in the respective studies, have limited predictive abilities when applied to tasks of predicting subsequent system behavior.

Finally, this work is related to the long tradition of learning ensembles for tackling various predictive modeling tasks in different ecological domains. Kocev and Džeroski et al. [16] present an ensemble method for learning habitat models of communities of organisms under different environmental conditions, using predictive clustering trees [17]. However, the ensemble methods presented in this paper are most closely related to those that tackle the problem of time-series forecasting. Knudby et al. [18] present novel approaches for modeling fish-habitat relationships using support-vector machines and tree ensembles for regression, where they aim at predicting fish species richness, biomass, and diversity from a range of habitat variables. Their main contribution is the extensive empirical study which identifies the most suitable machine learning method for short-term prediction since it aims at forecasting the next-time-point of fish-community concentrations. In contrast, our process-based ensembles aim at long-term (typically one year) prediction of systems behavior that concern periods with potentially indefinite ranges of time points.

## Materials and Methods

### Process-based models

The models of dynamic systems aim to describe the activities of the system components and the change of the system states over time. Mathematical equations serve as a powerful tool for achieving this aim, where the variables in the equations represent the state of the system components and the operators the interactions among components. Although this framework allows for adequate representation of dynamic systems, it forfeits the high-level information about the whys and hows of the modeled system’s behavior.

In essence, process-based models provide a conceptualization of the structure of the observed system, accompanied by modeling details that allow for their transformation to equations and therefore simulation. They tackle the task of describing dynamic systems from two aspects: qualitative and quantitative. From a qualitative aspect, a process-based model is a set of entities and processes. The entities represent the components of the observed system, which are involved in activities represented by the processes. From a quantitative aspect, a process-based model is interpreted as a set of differential and/or algebraic equations which can be used to simulate the behavior of the observed dynamic system. Process-based models encode both this low-level quantitative mathematical formalism, and a high-level qualitative description of the system.

**Fig 1:**
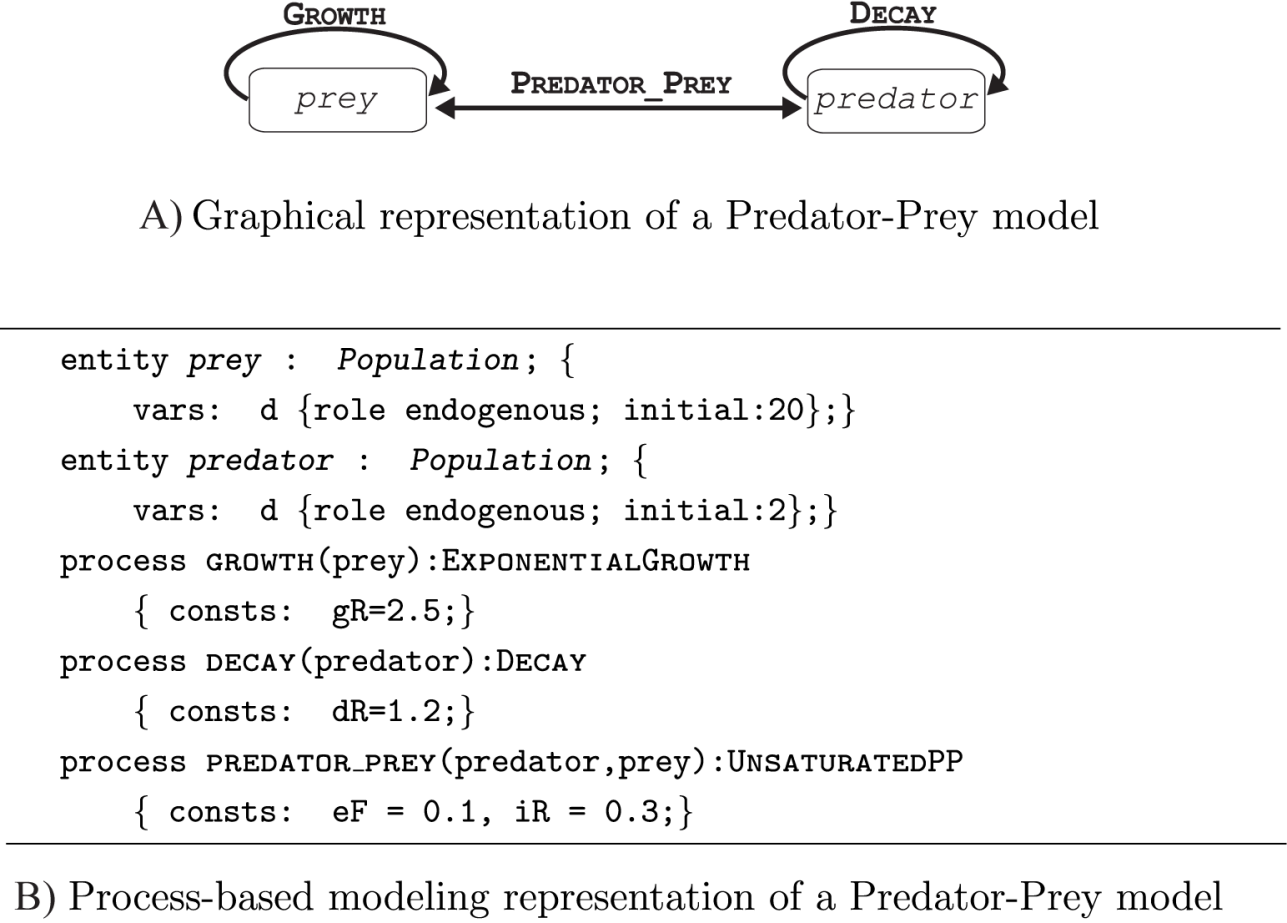
A simple Predator-Prey model and its process-based modeling representation. (A) A graphical representation of the entities (white boxes) and processes (black arrows) in a simple Predator-Prey model and (B) its process-based modeling representation.

Fig 1 gives both a graphical representation (Fig 1A) and a qualitative process-based representation (Fig 1B) of an example Lotka—Volterra Predator–Prey model. Notice how the PBM formalism represents the different processes/relations and entities/components involved in the model. For example, the predator_prey interaction is modeled as UnsaturatedPP and involves two entities *predator* and *prey* which are both part of the *Population* involved in the modeled system. Moreover, the growth and the decay of the *predator* and *prey* are modeled as ExponentialGrowth and Decay, respectively.

The entities and processes in Fig 1B represent the specific structure and dynamics of the particular system at hand. To elucidate such specific entities and processes, the process-based modeling approach uses entity and process templates, which serve as general placeholders for properties and definitions. The templates provide general modeling specifications for any instantiation to specific components or interactions, which in turn allows for a high-level qualitative conceptualization of a model to be translated into a low-level quantitative mathematical formalization which can be simulated.

Table 1, depicts the library of domain knowledge used to instantiate the Predator-Prey model from Fig 1B. The library organizes the templates in hierarchies. The *predator* and *prey* entities from the example are instances of the general template entity of *Population*. The entity templates incorporate group properties of the components of the modeled system. These properties include the variables (which change over time) and the constants related to the components of the modeled system, and their respective value constraints. For the variables, an aggregation function specifies how different process influences are combined for a particular entity. Take for example the *Population* template entity: it has one variable d which denotes density, and an aggregation function defined as summation, which means that, for the case of *prey*, the influences of both processes Growth and predator_prey on the variable d will be summed.

**Table 1:** Library of domain knowledge for modeling Predator-Prey dynamics.

template entity *Population*{vars: *d* {aggregation:sum, unit:“kg/m^3^”; range:<0,500>};\\density
template process Growth(*pop*: *Population*) { consts: gR {range:<0,5>}};
template process ExponentialGrowth:Growth {equations: td(pop.d) = gR * pop.d;}
template process LogisticGrowth:Growth {equations: td(pop.d) = gR * pop.d/(1 − pop.d/gR);}
template process Decay(*pop*: *Population*) { consts: dR{range:<0,2>}; equations: td(pop.d) = −dR * pop.d; }
template process Interaction(*pop1*:*Population*, *pop2*:*Population*){consts: iR {range: <0,2>}, eF {range: <0,1>};}
template process UnsaturatedPP: Interaction { equations: td(pop.d) = iR * eF * pop1.d * pop2.d, td(pop.d) = −iR * pop1.d * pop2.d; }
template process SaturatedPP: Interaction {consts: *sR*{range: <0,10>}; equations: td(pop.d) = iR * eF * pop1.d * pop2.d/(pop2.d+sR), td(pop.d) = −iR * pop1.d * pop2.d/(pop2.d+sR); }

The processes templates include specifications of the entity templates that interact, in terms of constants, algebraic and ordinary differential equations. The process templates are organized also into a hierarchy that defines the space of modeling alternatives. The particular process Growth, used in Fig 1B, is an instance of a more general process template Growth (Table 1), which is further instantiated to the process alternative— ExponentialGrowth (out of the two possible, i.e. ExponentialGrowth and LogisticGrowth).

Given such a library of domain knowledge, the task of learning process-based models takes two additional inputs, i.e., an incomplete model and measured data. Learning PBMs then proceeds in two phases: (1) instantiating the library of entity and process templates by using the incomplete model and (2) estimating the parameters in the resulting model structures to fit the measured data. Given the library of model fragments the former phase is formulated as a combinatorial search problem. Taking the incomplete model into account, one can instantiate the template entities and processes from the library into a set of specific components (entities and processes) to be included in the process-based model. The incomplete model represents modeling assumptions in terms of expected logical structure of the model, which limit the search space of the possible model structures, i.e., the combinations of model structures. Some of the combinations can be rejected as implausible, due to their inconsistency with the incomplete model in terms of presence or absence of certain processes. For example, an incomplete model of Predator-Prey dynamics can be obtained by removing the specific ExponentialGrowth and UnsaturatedPP processes and keeping their respective general templates. Together with the library presented in Table 1, this incomplete model results in 4 candidate model structures.

The latter task, i.e., estimating the model’s parameters is formulated as an optimization task. Each of the candidate model structures considered during the search task is compiled into a system of equations, for which a parameter estimation task is solved to obtain values of the model parameters that best fit the observed data. The objective function usually considered for such problems is minimizing the discrepancy between the model simulation and the observed system behavior.

The basic inductive process-based modeling algorithm, performs exhaustive search through a constrained space of candidate process-based models, limiting the number of processes in the model [14]. More advanced approaches, such as Lagramge2.0 [15] and HIPM [19], allow for more sophisticated hierarchical constraints on the allowed process combinations. The most recent PBM tool, the ProBMoT [2], allows for complete modeling, parameter estimation, and simulation of process-based models.

The first input to ProBMoT is a library of domain knowledge. The next input consists of the modeling assumptions formalized as an incomplete model of the observed system. The third and final input is a set of measured data. Based on the incomplete model and the library of domain knowledge, ProBMoT generates a set of model structures. For each of these structures, parameter estimation is performed so that they best fit the measurements. The parameter estimation process is based on the meta-heuristic optimization framework jMetal 4.5 [20] that implements a number of global optimization algorithms. For this purpose, ProBMoT implements a variety of error functions such as root mean squared error (RMSE), root relative squared error (RRSE) and weighted root mean squared error (WRMSE). Parameter estimation relies on model simulation, for which ProBMoT employs the CVODE ODE solver from the SUNDIALS suite [21].

Finally, the output of ProBMoT is a set of complete models sorted according to their performance, i.e., the difference error between the simulation and the measured data. In this study, we use ProBMoT as the learning algorithm for inducing process-based models, i.e., learning the constituents of the process-based ensemble models, which will be introduced in the next section.

### Ensembles of process-based models

Ensembles are an established method for improving the predictive performance of models in machine learning [5, 22]. An ensemble is a set of models (referred to as base models or ensemble constituents), that is expected to lead to a predictive performance gain over an individual model. In principle, any set of predictive models can be considered as an ensemble.

Once we have a set of predictive models, the question then arises as to how the individual predictions are to be combined into a single prediction. In traditional machine learning, this problem is tackled by using different combining schemes depending on the type of the base-models being aggregated. In the case of classification models that predict qualitative values, most often the predictions are combined by using different voting schemes.

The output of regression models that predict a single numeric value for a given input, can be combined by using the aggregation functions of average, weighted average and weighted median [23]. In this context, the output of an ensemble of process-based models resembles the one of time-series regression ensembles. However, in contrast to classical regression ensembles, the result of simulating an ensemble of process-based models is a whole trajectory instead of a single numeric value. Obtaining such a trajectory implies simulating each of the base models. This requires an initial value of the exogenous (state) variables and the trajectory of the exogenous (forcing) variables, which are then combined into an ensemble prediction. The different model simulations are combined time-point-wise: The combination is performed by well known methods usually applied in the case of numeric values, such as averaging, median, weighted average and weighted median [9].

When learning ensembles, the other important question is how to learn the constituent models of the ensemble. Methods for learning ensembles implicitly aim for diversity when learning the set of constituent models before aggregating their predictions. Based on how this diversity is achieved, we can distinguish two types of ensembles: heterogeneous and homogeneous. For learning heterogeneous ensembles, each of the base models is learned using a different learning algorithm (stacking [24]). On the other hand, in homogeneous ensembles, the individual base models are learned with the same learning algorithm, but from different samples of the training data, where the sampling variants include: sampling of the data instances (bagging [25], boosting [26]), sampling of the data features/attributes (random subspaces [10]) or both (bagging random subspaces [27], random forests [28]).

It has been theoretically and empirically shown that homogeneous ensembles, such as bagging and boosting, perform well for classification and regression problems [5, 28–30]. However, these methods, that sample data instances, can often be ineffective when the training data is relatively homogeneous. Additionally, when the dimensionality of the data (the feature space) is very high, learning such ensembles can be very ineffective and computationally complex.

The base level learning algorithm in this study is ProBMoT, and here we aim at learning homogeneous ensembles of process-based models. In the realm of process-based models, ensembles learned by sampling data instance, i.e., bagging and boosting, have demonstrated improved performance over individual models [8, 9], at the cost of increased computational complexity. In the continuation of this section, we outline the methods for sampling data instances in the context of process-based modeling. We then shift our focus to a method for learning ensembles of process-based models via sampling data features, i.e., sampling domain knowledge.

#### Sampling data instances for learning ensembles of process-based models

Bagging (bootstrap aggregation) refers to an approach, developed by Breiman [25], for constructing ensembles via bootstrap sampling with aggregation. This is one of the first and simplest ensemble learning methods, where data instances are uniformly sampled with-replacements to generate random samples (bootstrap replicates) of the training data, consequently used to learn a set of ensemble constituents. The learned base models are then combined by averaging their output (in the case of regression) or by voting (in the case of classification). Bagging ensembles successfully overcome the over-fitting problem most often when constitute instable models, i.e. models that can change dramatically even for small changes in the training data. However, they are not that accurate when are constructed with stable models.

More sophisticated ensemble methods, such as boosting, obtain ensemble predictions by combing “imperfect” predictions made by base models learned on different distributions of the training data. The most notable implementation of boosting is the AdaBoost algorithm, proposed by Freund and Schapire [31], originally developed for tackling classification tasks, and later adapted by Drucker [23] for combining regressors. Like bagging, AdaBoost also re-samples the training data: However, instead of treating all instances equally (as in bagging), it prioritizes the more informative ones (i.e., those where large errors are currently made) for each subsequent iteration. Even though AdaBoost is very successful in tackling the over-fitting problem on a variety of tasks, its performance is prone to noise in the training data which can lead to lower performance as compared to single models and to other ensemble methods such as bagging [5].

For the task of bagging and boosting ensembles of process-based models, the candidate base models are learned from different samples of the measured data. The notable difference from bagging and boosting in the context of regression is that, in our case, the data instances have a temporal ordering, that has to be retained in each sample of the data. A detailed specification of how bagging and boosting are implemented in the context of process-based modeling can be found in the study by Simidjievski et al. [9].

#### Sampling domain knowledge for learning ensembles of process-based models

The random subspace method (RSM) is a homogeneous ensemble method developed by Ho [10], which constructs different variants of the training data by sampling the feature space. Each ensemble constituent is learned on all data instances and a subspace of the original feature space. The predictions of the learned base models are then combined via standard combining schemes for classification and regression, i.e., voting schemes and averaging techniques, respectively. The RSM has been reported to perform well for problems where the data dimensionality is very high or when there is a certain redundancy in the feature space [32].

In the context of learning ensembles of process based models, we can think of the feature space as being defined by the model components instantiated from the process templates. This space of components is determined by the number of process alternatives defined in the library of domain knowledge. Therefore, generating random samples of the feature space used in the traditional RSM, is analogous to generating random samples of the library of domain knowledge. The approach presented in this paper learns ensemble constituents from the whole data set using samples of the knowledge library. This is in contrast with bagging and boosting, where ensemble constituents are being learned on data samples using the same knowledge library or feature space.

The procedure for learning ensembles of process-based models via library sampling is presented in Algorithm 1. The procedure LS() takes five inputs: a library of domain knowledge (*lib*), a dataset consisting of training data (*D*_*T*_) and validation data (*D*_*V*_), an incomplete model (*incompleteModel*), a boolean variable (*allowDuplicates*), and an integer *k* denoting how many base models are to be generated. The output is a set of process-based models denoted with *Ensemble*.

**Algorithm 1** Learn ensembles of process-based models via library sampling

 1: **procedure** LS(*lib*, {*D*_*T*_, *D*_*V*_}, *incompleteModel*, *allowDuplicates*, *k*)

 2: **returns** *Ensemble*

 3:    Ensemble ← ⌀      ▹ set of base models

 4:   **do**

 5:    *lib*_*S*_← SAMPLE(*lib*)     ▹ randomly sample the library *lib*

 6:    *modelList*_*i*_← PROBMOT(*lib*_*S*_, *D*_*T*_, *incompleteModel*)

 7:    *bestModel*_*i*_← RANK(*modelList*_*i*_, *D*_*V*_)

 8:    *β*_*i*_←CONFIDENCE(*bestModel*_*i*_, *D*_*V*_)    ▹ presented in Algorithm 2

 9:    **if** *allowDuplicates* **then**

 10:     *Ensemble* ← *Ensemble*⋃ (*bestModel*_*i*_, *β*_*i*_)

 11:    **else if** *bestModel*_*i*_ ∉ *Ensemble* **then**

 12:     *Ensemble* ← *Ensemble*⋃ (*bestModel*_*i*_, *β*_*i*_)

 13:    **end if**

 14:   **While** SIZE(*Ensemble*)≠*k*

 15: **end procedure**

**Algorithm 2** Calculating confidence

 1: **function** confidence(*model*, *D*) **returns** *β*

 2:   let $\hat{y}$      ▹ simulated system variable *y*

 3:   let *y*      ▹ measured system variable *y*

 4:   $\hat{y}\leftarrow$ SIMULATE(*model*, *D*)

 5:   $maxDisc\leftarrow \sup{\left(|y_{t}-{\hat{y}}_{t}|\right)}^{2}$     ▹ calculate max discrepancy between measurements *y* and simulation $\hat{y}$, where *t* = 0..*N* and *N* is number of time-points in D

 6:   $\bar{L}\leftarrow \sum_{t=0}^{N}\frac{{|y_{t}-{\hat{y}}_{t}|}^{2}}{maxDisc}$       ▹ calculate average loss

 7:   $\beta \leftarrow \frac{\bar{L}}{1-\bar{L}}$       ▹ calculate confidence

 8: **end function**

For the task of sampling the library (SAMPLE(*lib*), line 5 in Algorithm 1) the process alternatives are randomly sampled (included or excluded) from the original library. The sampling algorithm takes as input the complete library and considers all the process templates defining more than one modeling choice. In turn, for each process template considered, it takes a random sample of the available modeling choices to be included in the sampled library. Note that the library sampling does not assume a uniform distribution of samples: the probability of a library sample is proportional to the size of the induced space of candidate models. In particular, the probability of a library sample *lib*_*S*_ of the whole library *lib* equals

$$\begin{array}{c} P\left({lib}_{S}\right)=\frac{|L_{S}|}{\sum_{L_{i}\in P\left(L\right)}\left|L_{i}\right|}\;, \end{array}$$

where *L* and *L*_*S*_ ⊆ *L* correspond to the sets of candidate models induced by *lib* and *lib*_*S*_ (for a given incomplete model specification), respectively. Moreover, |⋅| denotes set cardinality and P(L) denotes the powerset of *L*, i.e., the set of all the possible subsets of *L*. For example, there are nine samples of the library from Table 1: one that generates four candidate models, four that induce two candidate models each, and four resulting in one candidate model each. The last four library samples are less likely selected (the probability of selecting each is 1/16) than the other five samples (1/4 for the first one, and 1/8 for each of the remaining four).

The process-based modeling algorithm PROBMOT(), (line 6 in Algorithm 1), takes as input the sample of the library of domain knowledge *lib*_*S*_, time-series measurements of the observed dynamic system *D*_*T*_, and an *incompleteModel* representing the modeling assumptions made by the modeler. The output of PROBMOT() is a list of process-based models, which is afterwards sorted according to their performance (line 7 in Algorithm 1). The output of the function RANK() (line 7 in Algorithm 1), i.e., the highest ranked model from each modeling task *i* (out of *k*) denoted as *bestModel*_*i*_, becomes an ensemble constituent in the output *Ensemble*. The ranking can be based on the performance on a separate validation data set *D*_*V*_ or on the training sample (if *D*_*V*_ = = *D*_*T*_).

Each ensemble constituent is paired with its own confidence *β*. The calculation of the CONFIDENCE() (Algorithm 2) function takes 2 inputs: the highest ranked model returned by ProBMoT and a data set *D*. First, the *model* is simulated on the data set *D* resulting in a trajectory $\hat{y}$. Based on the error at each time point in the trajectory, an average loss $\bar{L}$ is calculated for the *model* (line 6 in Algorithm 2). From this loss, a confidence measure *β* is calculated, where low values of *β* denote high confidence. The *β* coefficient is an indicator of the performance of the base model and is used in the process of simulating the ensemble, i.e., combining the simulations of the constituent models into an overall ensemble prediction when weighted combining schemes are considered (i.e., Weighted Average and Weighted Median).

Given the fact that the method always takes as input the same original library, there is a high probability of learning and choosing identical models from different library samples, thus filling the ensemble constituent set with multiple copies of the same model. To account for this, the method incorporates two different alternatives for generating the ensemble constituent set, i.e., with and without duplicates. For the former, referred to as *Library Sampling with Duplicates*, duplicates are allowed in the constituent set (line 9 in Algorithm 1). *k* library samples are generated (with *k* denoting number of ensemble iterations), and the best model out of each modeling task is chosen to be an ensemble constituent, regardless of whether that particular model was already in the constituent set or not. For the latter, referred to as *Library Sampling without Duplicates*, to incorporate more diversity in the ensemble, the method generates library samples (and performs modeling tasks) until the resulting ensemble contains *k* distinct constituents (line 11 in Algorithm 1).

### Experimental Setup

In this section, we present the setup of the experiments used to evaluate the predictive performance of the ensembles of process-based models. We first introduce the data sets to be used in the experiments, then we briefly describe the two other ProBMoT inputs, the library of domain knowledge and the modeling assumptions. Next, we give an overview of the parameters of the algorithm for learning ensembles with bagging and boosting, used in the last set of experiments. Finally, we finish the section with the metrics used to measure the performance of process-based models and ensembles thereof.

#### The data

The performance of the proposed method for learning ensemble is evaluated on several tasks of modeling population dynamics in three aquatic ecosystems: Lake Bled in Slovenia, Lake Zurich in Switzerland and Lake Kasumigaura in Japan.

Lake Bled is located in the Julian Alps in north-western Slovenia and occupies an area of 1.4 *km*^2^, with a volume of 0.0257 *km*^3^, a maximum depth of 30.1 *m* and an average depth of 17.9 *m*. The measurements, performed by the Slovenian Environment Agency, consist of physical, chemical and biological data for the period from 1996 to 2002. All the measurements were performed once a month and depth-averaged for the upper 10 *m* of the lake.

Lake Zurich is located in the south-western part of the canton of Zurich in Switzerland. It has an average depth of 49 m, a volume of 3.9 *km*^3^ and a surface area of 88.66 *km*^2^. The data comprise measurements, performed by the Water Supply Authority of Zurich in the period from 1996 to 2002. They include profiles of physical, chemical and biological variables from 19 different sites, weight averaged to the respective epilimnion (top 10 *m*) and hypilimnion (bottom 10 *m*) depths. To obtain daily approximations, the data for both lakes were interpolated with a cubic spline algorithm and daily samples were taken from the interpolation [4, 33].

Lake Kasumigaura, located 60 *km* to the north-east of Tokyo, Japan, has an average depth of 4 *m*, a volume of 0.848*km*^3^, and a surface area of 220*km*^2^. The dataset comprises monthly measurements, taken in the period from 1986 to 1992. Similarly, to obtain daily approximations, the measurements were interpolated using linear interpolation and daily samples were taken from the interpolation [34].

In this paper, the same structure of a population dynamics model is used in all three aquatic ecosystems. It includes a single equation (ODE) for a system variable representing the phytoplankton biomass (measured as *chlorophyll-a* in Kasumigaura). The exogenous variables include the concentration of the zooplankton *Daphnia hyalina* (where available, i.e., for Bled and Zurich only), dissolved inorganic nutrients of nitrogen, phosphorus, and silica (measured as *ammonia* in Kasumigaura), as well as two input variables representing the environmental influence of water temperature and global solar radiation (light).

In the experiments, we use fifteen data sets, which are subsets of the above-mentioned measured data from the aquatic ecosystems in the three lakes. For each aquatic ecosystem, the original data set is split into seven single-year data sets. Five of these are used (one at a time) for training the ensemble constituents. From the remaining two, one is used for validating the models in the process of selecting the ensemble constituents, and one to measure the predictive performance of the learned process-based models and ensembles thereof. Therefore, fifteen learning experiments are performed; in each, we take a single-year training data set, learn a model using the train and the validation data sets, and test the predictive performance of the learned model on the test data set. In Table 2, which reports the experimental results, the experiments are labeled with the labels B1–B5, K1–K5 and Z1–Z5 corresponding to the training data set using in the experiment, where, e.g., K3 denotes the Lake Kasumigaura data set for the third year (i.e., 1988).

**Table 2:** Comparison of the predictive performance of a single model with that of ensembles of PBMs learned with library sampling, bagging and boosting.

Case	SingleModel	Library Sampling	Bagging	Boosting
B1	5.184 (4)	1.103 (3)	1.073 (2)	**1.065** (1)
B2	**0.938** (1)	1.143 (4)	1.085 (3)	0.947 (2)
B3	1.042 (4)	**0.794** (1)	0.968 (3)	0.941 (2)
B4	2.840 (4)	**0.750** (1)	0.760 (3)	0.791 (2)
B5	0.842 (2)	0.858 (3)	0.859 (4)	**0.698** (1)
K1	7.730 (4)	0.756 (2)	**0.736** (1)	0.925 (3)
K2	328.138 (4)	**0.878** (1)	1.429 (2)	1.642 (3)
K3	0.932 (4)	**0.827** (1)	0.867 (2)	0.923 (3)
K4	0.777 (3)	0.800 (4)	0.772 (2)	**0.705** (1)
K5	0.792 (4)	**0.732** (1)	0.734 (2)	0.781 (3)
Z1	**0.744** (1)	0.797 (3)	0.777 (2)	0.890 (4)
Z2	1.323 (4)	0.948 (3)	**0.883** (1)	0.893 (2)
Z3	29.463 (4)	**0.881** (1)	0.934 (2)	1.001 (3)
Z4	27.593 (4)	1.011 (3)	**0.964** (1)	0.982 (2)
Z5	1.489 (4)	1.130 (2)	**1.113** (1)	1.403 (3)

Note that, we follow the traditional experimental setup used in ecological modeling, where predictive models for a particular aquatic ecosystem are learned and tested on data from that same ecosystem. This is due to the fact that many environmental variables, corresponding to overall properties of the observed system (such as depth, volume and terrain configuration), are assumed to be constant in the modeling process. Additionally, some of the entities (variables) and ecological processes typically differ between ecosystems. Thus, models learned under this assumption can not be directly used in the context of other ecosystems.

#### The library of domain knowledge

In the performed experiments, we use the library of domain knowledge for modeling population dynamics in aquatic ecosystems, presented in čerepnalkoski et al. [2]. This library is based on the previous work of Atanasova et al. [35]. The library of domain knowledge, combined with the modeling assumptions, results in 18144 candidate models for Lake Kasumigaura and 27216 candidates for the other two lakes.

#### ProBMoT parameter settings

ProBMoT implements the Differential Evolution (DE) [36] method for parameter estimation. For the experiments performed in this paper, the DE parameters were set as follows: a population size of 50, strategy *rand/1/bin*, differential weight (*F*) and the crossover probability (*Cr*) of 0.6. The limit on the number of evaluations of the objective function is one thousand per parameter. For simulating the ODEs, the CVODE simulator is used with absolute and relative tolerances set to 10^−3^.

#### Learning ensembles of process-based models with bagging and boosting

To properly assess the predictive performance of the proposed method for learning ensembles of process-based models, in our last set of experiments we compare it to the state-of-the-art methods for learning ensembles, i.e., bagging and boosting. Following the finding of Simidjievski et al. [9] the ensembles are learned with both bagging and boosting include 25 constituents, which are chosen based on their performance on a separate validation set and combined by averaging their predictions. These settings were chosen by following the same experimental setup that we use in this paper to select the appropriate design choices for learning ensembles with library sampling.

Note, however, an important difference between the setup of the bagging and boosting experiments and the experiments with library sampling. In the latter, we use the whole library of domain knowledge as described previously in subsection *The library of domain knowledge*. The use of this library is prohibitive for bagging and boosting, due to the high computational complexity considering the large space of candidate models in each learning iteration. To address this issue, we use a simplified version of the original library that results in 320 candidate model structures for Lake Kasumigaura and 128 candidates for the other two lakes. We have prepared the simplified library carefully, omitting only modeling alternatives (process templates) that are rarely observed to be among the top-ranked models in the single-model experiments with ProBMoT. The issue of computational complexity of learning ensembles of PBMs is further discussed in the subsection *The computational complexity of learning ensembles of PBMs* of the section *Results*.

#### Performance evaluation metrics

To evaluate the predictive performance of a given model *m*, we use the measure of relative root mean squared error (*ReRMSE*) [28], defined as:

$$\begin{array}{c} ReRMSE(m)=\sqrt{\frac{\sum_{t=0}^{n}{\left(y_{t}-\hat{y_{t}}\right)}^{2}}{\sum_{t=0}^{n}{\left(\bar{y}-\hat{y_{t}}\right)}^{2}}}, \end{array}$$ (1)

where *n* denotes the number of measurements in the test data set, *y*_*t*_ and $\hat{y_{t}}$ correspond to the *measured* and *predicted* (predictions are obtained by simulating the model *m* on the test set) value of the system variable *y* at time point *t*, and $\bar{y}$ denotes the mean value of *y* in the test data set. Note that the usual root mean squared error observed here, is relative to the standard deviation of the system variable in the test data, thus allowing us to compare the errors of models for different system variables with measured values on different scales (e.g., phytoplankton in different lakes).

#### Statistical comparison of performance

We observe and compare the predictive performance (in terms of *ReRMSE*) of the models learned using different algorithms on the 15 data sets. To properly assess the significance of the differences between the performances of models obtained with different algorithms, we follow the standard statistical procedure recommended by Demšar [37]. We use the corrected [38] Friedman test [39], followed by two post-hoc tests: the Nemenyi test [40] and the Bonferroni-Dunn test [41]. A positive outcome of the Friedman test indicates difference between the performances of the different algorithms considered. After the completion of the Fridman test, we proceed with performing post-hoc tests to identify which differences are statistically significant.

**Fig 2:**
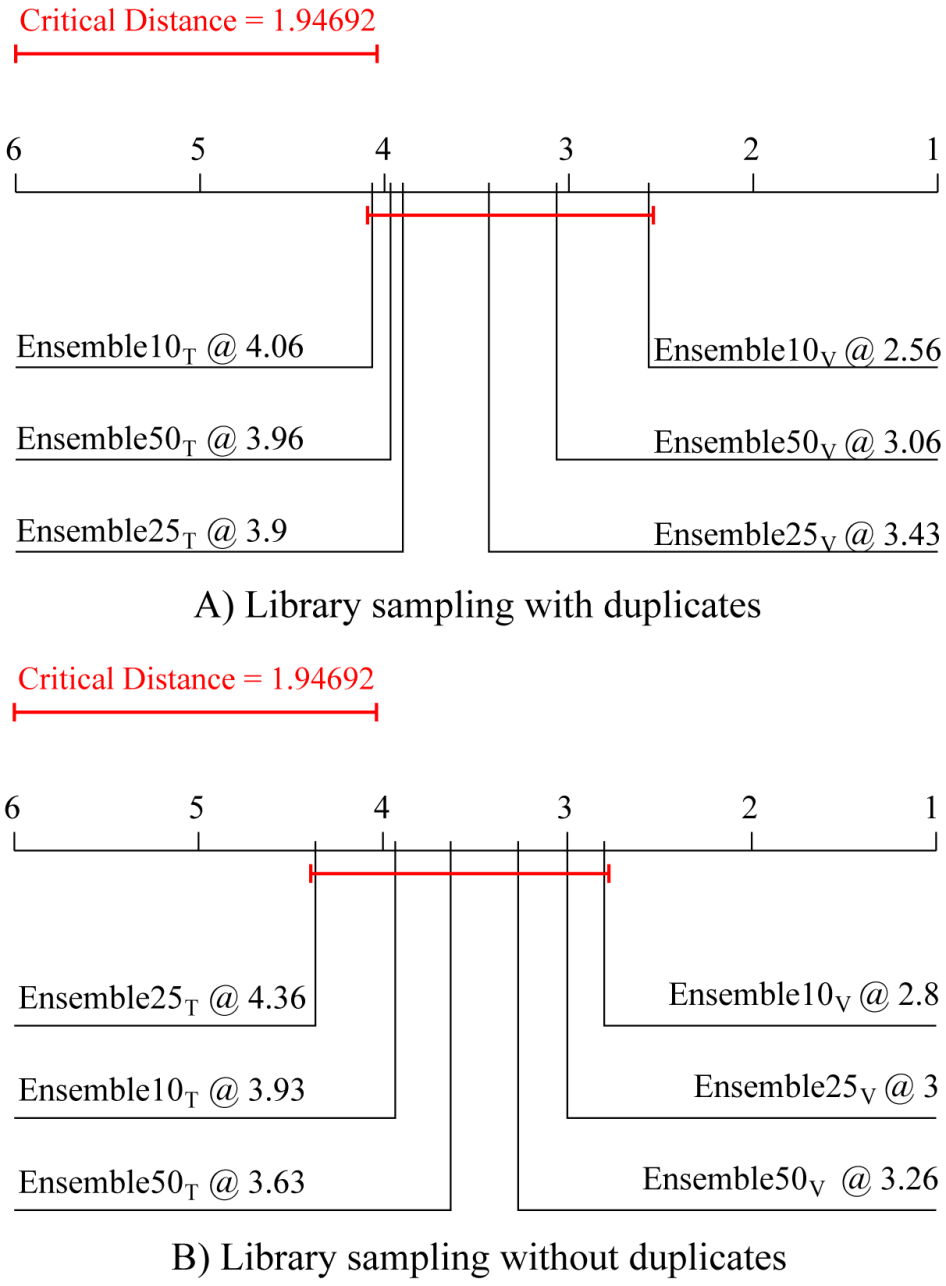
Comparison of the predictive performance in terms of average ranks of the two library sampling methods with different methods for choosing ensemble constituents. Average ranks of ensembles with 10, 25 and 50 constituents combined by averaging and selected differently (based on their train (subscript T) or validation (subscript V) performance). The average ranks refer to the predictive (testing) model performance averaged over the 15 experimental data sets, separately for the case of Library sampling (A) with and (B) without duplicates.

**Fig 3:**
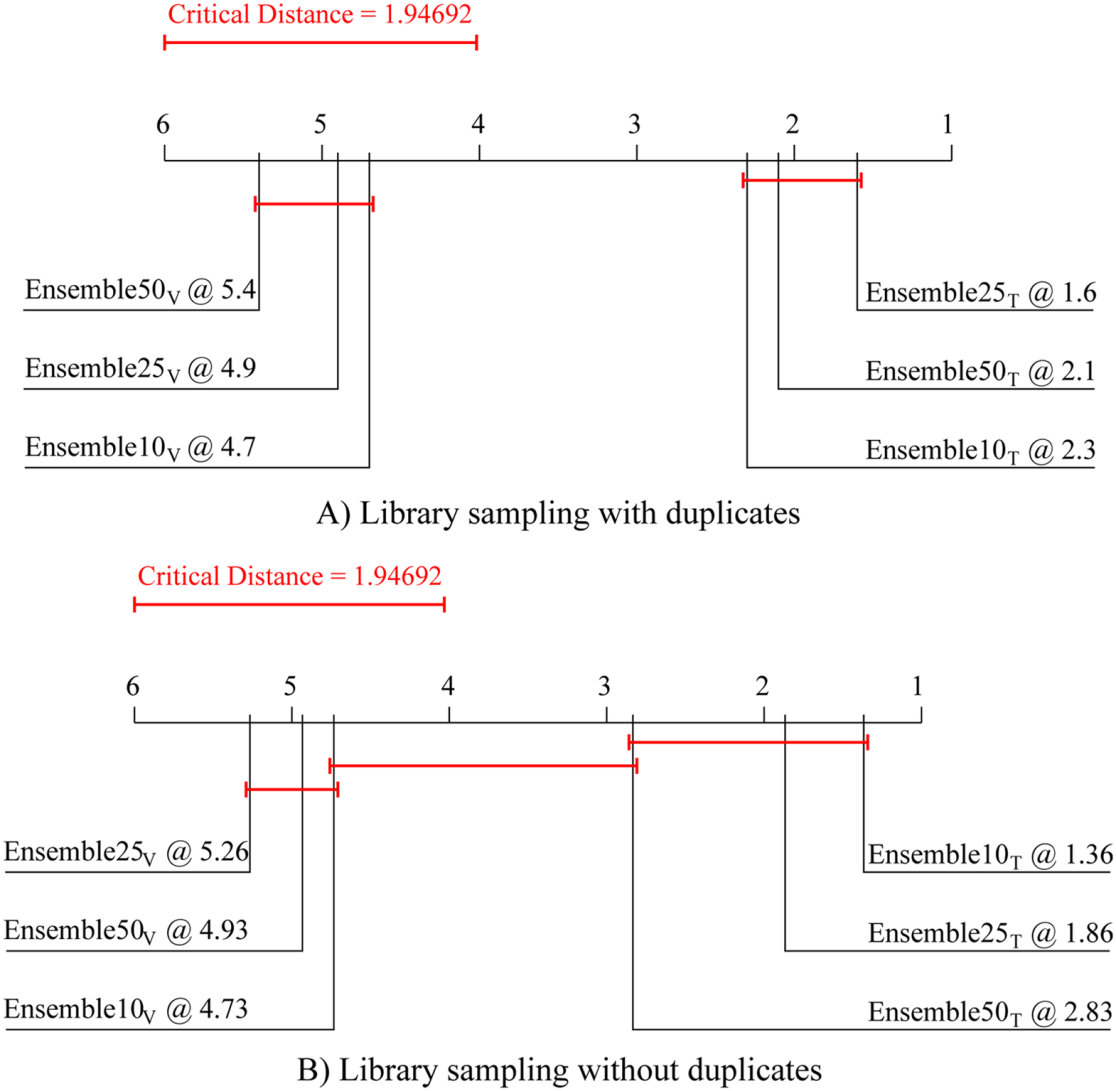
Comparison of the average ranks for descriptive performance (on training data) of the two library sampling methods with different methods for choosing ensemble constituents. Average ranks of ensembles with 10, 25 and 50 constituents combined by averaging and selected differently (based on their train (subscript T) or validation (subscript V) performance). The average ranks refer to the descriptive model (training) performance averaged over the 15 experimental data sets, separately for the case of Library sampling (A) with and (B) without duplicates.

**Fig 4:**
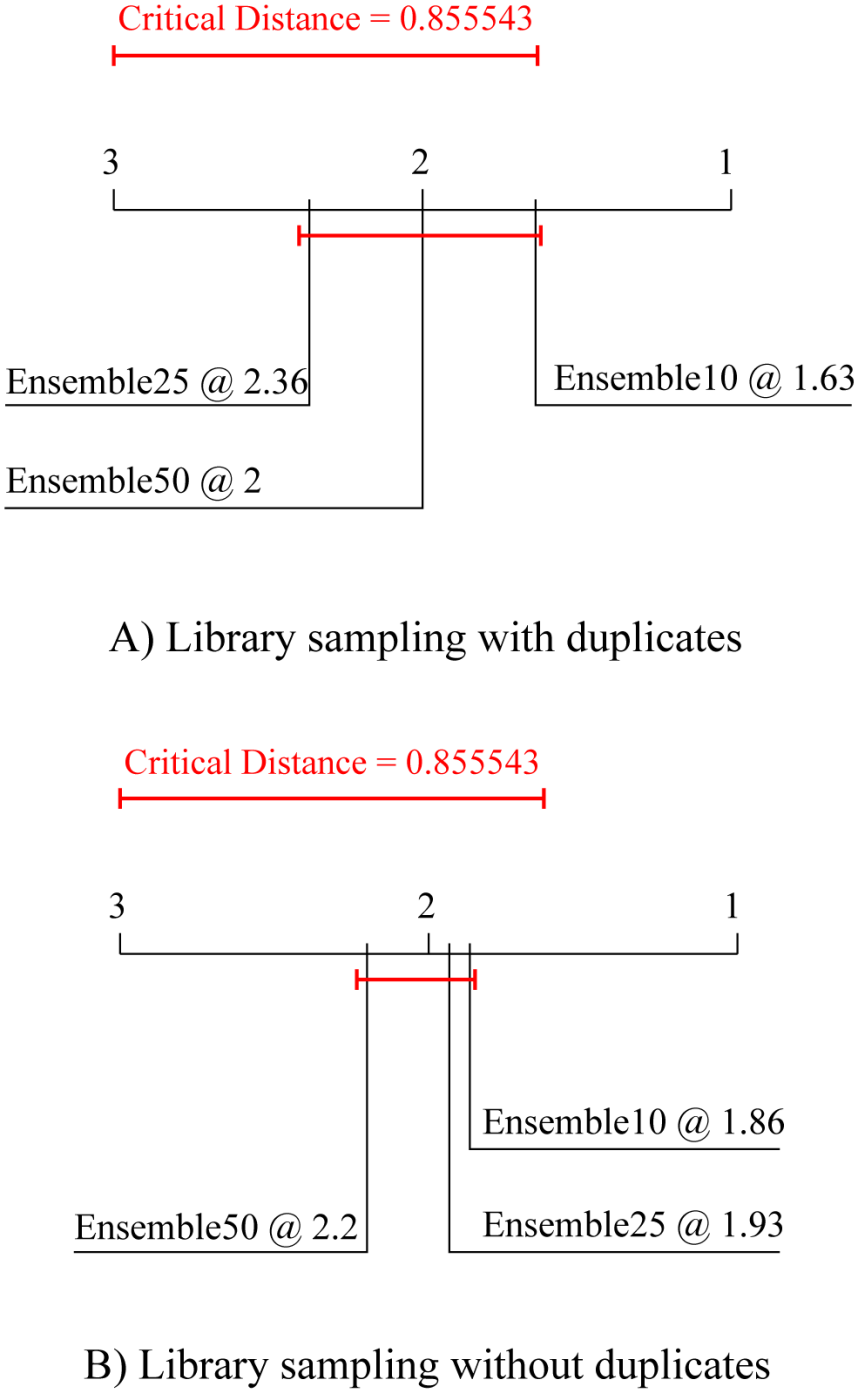
Comparison of the average ranks for predictive performance of the two library sampling methods with different ensemble sizes. Average ranks of ensembles that include 10, 25, and 50 base models in terms of predictive performance averaged over the 15 experimental data sets for Library sampling (A) with and (B) without duplicates.

**Fig 5:**
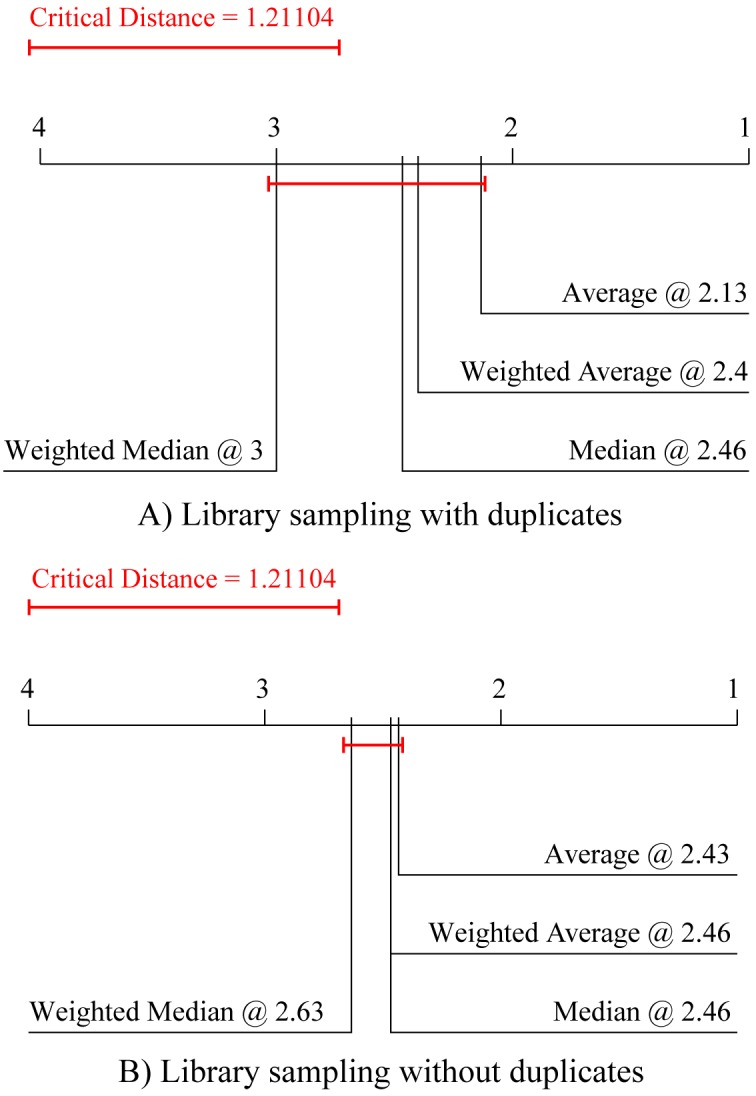
Comparison of the average ranks for predictive performance of the two library sampling methods with four combining methods. Average ranks of the four methods for combining the simulations of base models (average, weighted average, median and weighted median) in terms of predictive performance averaged over the 15 experimental data sets for Library sampling (A) with and (B) without duplicates.

**Fig 6:**
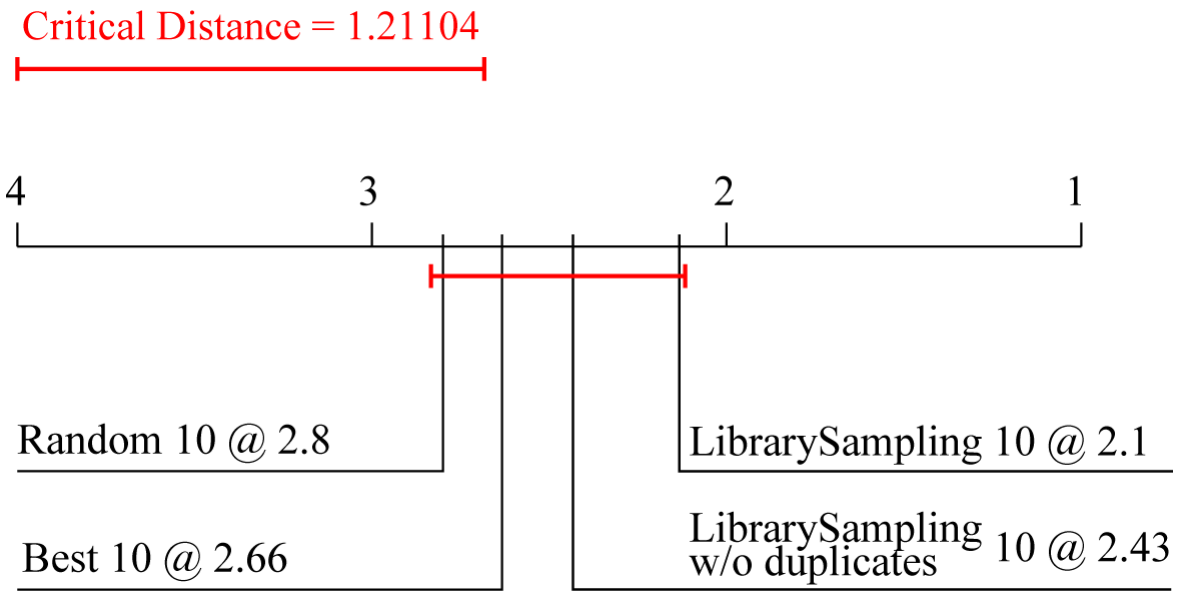
Comparison of the average ranks for predictive performance of the two library sampling methods to two alternative ensemble approaches. Average ranks of four different types of ensembles with 10 constituents combined by averaging. The constituents are: constructed via library sampling with and without duplicates, the 10 best models learned from the complete library, and 10 random models learned from the complete library. The average ranks of predictive performance are computed over the 15 data sets.

The first post-hoc test, i.e, the Nemenyi post-hoc test, computes the critical distance between the algorithm ranks at a given level of statistical significance (in this paper, the significance level threshold is set at 95%, *p* = 0.05). Only differences of the average ranks larger than the critical distance are considered significant; for those, we can claim that one algorithm outperforms (i.e., performs significantly better than) the other. This test is performed to obtain an assessment of the relative performance of the methods considered. In this paper, the Nemenyi post-hoc test is employed for comparison of different design decisions for the newly proposed method. The results of the Friedman-Nemenyi tests are depicted by average rank diagrams (as in Figs 2–6), where the critical distance is shown as a solid red line.

**Fig 7:**
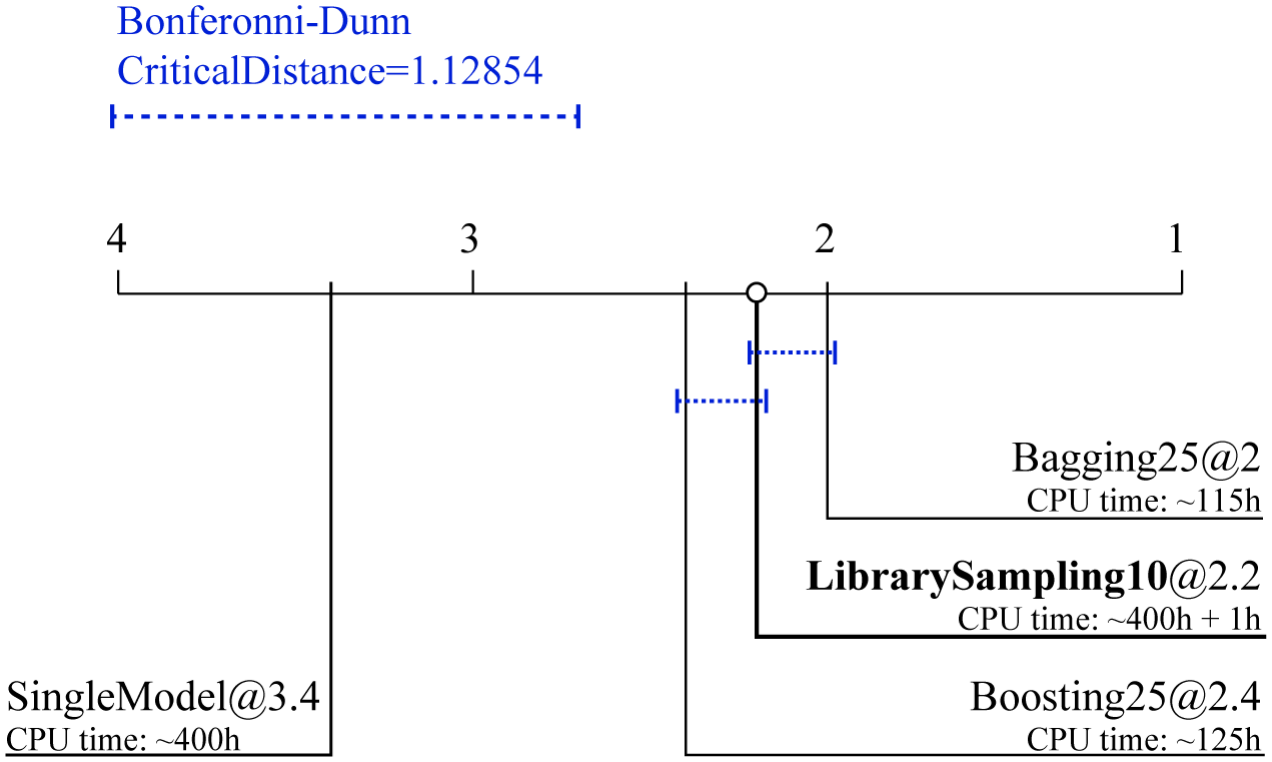
Comparison of the average ranks for predictive performance of the library sampling method to learning single models, bagging and boosting. Average ranks of the ensembles constructed by library sampling with 10 constituents combined by averaging to the performance of: a single model and two types of ensembles combined by averaging constructed by bagging and boosting (with 25 constituents). The ranks of the models in terms of their predictive performance and the average CPU times for learning them are averaged over the 15 data sets.

The second post-hoc test, i.e., the Bonferroni-Dunn post-hoc test, is performed to test how a proposed method performs in a comparison to other methods. This test is similar to the Nemenyi test, where a critical distance between the algorithm ranks is computed at a given level of significance (in this paper the significance level threshold is at 95%, *p* = 0.05), denotes how one method (i.e, an ensemble learned using the library sampling method) compares to the other existing methods for constructing ensembles of process-based models (i.e., bagging, boosting) and a single model, in terms of predictive performance. The results of the Friedman-Bonferroni-Dunn test is depicted by the average rank diagram (as in Fig 7), where the critical distance is shown as a dashed blue line.

## Results

In this section, we present and discuss the results of the empirical evaluation. Given the fact that the method for learning ensembles with library sampling is novel, we first identify the most suitable design choices within the algorithm. We investigate what are the optimal choices of method for selecting the ensemble constituents, number of ensemble constituents, and combining method. The optimal choices are identified for both library sampling alternatives (with and without duplicates).

After making the design choices, we compare the predictive performance of the library-sampling ensembles with the performance of baseline ensembles, state-of-the-art bagging and boosting ensembles, and single models. The two baseline ensembles consist of the top-ten and ten randomly selected process-based models. Finally, we perform a comparative analysis of the computational complexities of different methods for learning ensembles of process-based models.

### Design choices for the algorithm for learning library-sampling ensembles

The first design choice in an algorithm for learning ensembles of PBMs is related to the way of choosing the ensemble constituents. As previously outlined, the highest ranked model of each ensemble iteration is selected to be an ensemble constituent. The standard approach of ProBMoT to ranking candidate models is with respect to their performance on the training set. However, to avoid overfitting, these models can be re-ranked according to their performance on a separate validation data set. These experiments are performed using ensembles with 10, 25 and 50 constituents whose predictions are combined by averaging.

Fig 2 summarizes the performance comparison of the methods for choosing the base models to be included in the ensembles. The upper diagram (Fig 2A) depicts the results of the Friedman-Nemenyi test for library sampling with duplicates, and the lower diagram (Fig 2B) for library sampling without duplicates. In both cases, choosing the base models based on their ranking on a separate validation set leads to better ensemble performance. However, in both cases, this improvement is not significant.

Earlier we conjectured that choosing ensemble constituents based on their performance on the training data might lead to overfitting. Fig 3 confirms this conjecture: In both cases (Fig 3A and 3B), ensembles comprising base models selected based on the performance on the training data exhibit significantly better descriptive/training performance than the ones selected based on their performance on a separate validation dataset. This is in-line with our previous findings for bagging and boosting [9], where the ensembles with constituents chosen on the bases of their training performance demonstrate a clear case of overfitting—while having significantly better performance on the training data these ensembles have worse predictive performance on unseen data.

Next, we focus on choosing the optimal number of base models in the ensembles of process-based models. To this end, we compare the predictive performance of ensembles consisting of 10, 25 and 50 base models, whose predictions are combined by averaging. The Friedman-Nemenyi diagram, presented in Fig 4, shows that the ensembles containing 10 ensemble constituents, for both types of ensembles (Fig 4A and 4B), lead to the best performance. Note however, that the observed difference in performance is not statistically significant.

Finally, we focus on choosing the most appropriate method for combining the simulations of the base models in the ensemble. We compare the performance aggregations of four methods that are often used in ensembles of regression models: the average, weighted average, median and weighted median methods. Fig 5 depicts the comparison of the average ranks for these methods both for library sampling with and without duplicates (Fig 5A and 5B, respectively), containing 10 constituents. It can be observed that in both cases the simple average method for aggregation performs (not significantly) best.

In summary, based on the performed experiments we make the following design decisions related to learning ensembles of process-based models with library sampling. First, the ensemble constituents are chosen based on their performance on a separate validation set, as they exhibit better performance than the ones chosen based on their performance on the original training data set. Second, the ensembles should consist of relatively small number(10) of base models whose predictions should be combined by using the simple average method. Although the latter conclusion is based on results which are not statistically significant, it can be justified by following the parsimony principle. In all further experiments, we used these algorithm settings for learning ensembles for library sampling with and without duplicates.

### Predictive performance of the library-sampling ensembles

Here we focus on testing our central hypothesis that the ensembles learned with library sampling improve the predictive performance of process-based models. However, to properly assess the performance of such ensembles, and whether/how it is related to the sampling of the library, we first compare the performance of both alternatives of library sampling to two baseline types of ensembles. The first baseline ensemble is comprised of the 10 best performing models learned using the complete library, combined by averaging. We refer to this ensemble as *Best10*. The second baseline ensemble is comprised of 10 randomly chosen models, also learned on the complete library and combined by averaging. We refer to this ensemble as *Random10*.

Fig 6 depicts the Friedman-Nemenyi comparison of the average ranks of the two library-sampling ensembles and the two baseline ensembles. It can be seen that the ensemble with library sampling with duplicates performs best, followed by the one which uses library sampling without duplicates. Note, however, that the test shows no significant difference in performance among the four approaches.

Given that the experiments performed so far did not show any substantial difference in the performance between the two alternatives for library sampling, we further investigated their structure. We found that the constituent set with 10 elements of the ensemble learned by library sampling with duplicates has on average 85% unique base models. Moreover, the method which samples the library without duplicates required on average 4 more iterations (per dataset) for learning an ensemble with 10 constituents. Given the results in Fig 6, and this insight into the structure of the ensembles, we follow the parsimony principle once again, and consider the method of library sampling with duplicates to be the more efficient and effective alternative when learning such ensembles.

In our last set of experiments, we assess the predictive performance of the method for learning ensembles via library sampling with the optimal design choices determined above. We compare its performance to the performance of a single model learned on the complete library, and ensembles learned using a reduced version of the library by the two state-of-the-art methods in process-based modeling, i.e., bagging and boosting with 25 constituents each and combined by averaging.

Fig 7 depicts the Friedman-Bonferroni-Dunn comparisons of the average ranks across the fifteen data sets. The results of the test show that the proposed ensembles with library sampling *significantly* outperform single process-based models. This result supports the central hypothesis of our paper that such ensembles improve the predictive performance over single process-based models. Note, however, that their predictive performance is slightly (not significantly) worse than bagging ensembles, and slightly (not significantly) better than boosting ensembles.

Fig 7 also presents the CPU time needed for learning all the ensembles and the single model. On average, learning ensembles with the library sampling method takes as much time as learning a single process-based model (≈400 *h*). Note, however, that even though the remaining two methods of bagging and boosting take far less time than the previous methods, (≈115 *h* and ≈125 *h*, respectively), they are learned from a reduced library. After a further investigation, which involved learning a single model on the smaller library (that took 4.5 to 5*h*), we estimate that it would take ≈9200 *h* and ≈10000 *h*, for bagging and boosting of process-based models using the original libraries, respectively. This would result in substantially (by a factor of ≈25) worse computational efficiency as compared to learning library-sampling ensembles.

So far, our comparison of the methods focused on their average ranks, which do not reveal the actual performance of the models obtained with the different methods. Table 2 reports these performances of the single process-based models and the three ensembles of PBMs learned with library sampling, bagging and boosting. Note that the results reported in the table confirm the superiority of ensembles to a single model. The ensemble methods are far more robust than single models, which severely under-perform in 6 out of the 15 cases: in these 6 cases, the single models have a ReRMSE of over 2. In the two cases where the single models outperforms the ensembles (B2 and Z1), the difference in performance when compared with the top-ranked ensemble method for the particular case is minor. On the other hand, ensemble methods can not be differentiated in terms of robustness: all three methods for learning ensembles of PBMs are equally robust. The differences in performances among the models obtained with the different ensemble methods are minor and often negligible. The relative winner is the proposed algorithm for library sampling, that outperforms the other two methods in 6 out of 15 cases. In terms of predictive performance, the method of library sampling is competitive and even slightly better than the state-of-the-art ensemble methods of bagging and boosting PBMs, leading to more accurate predictive models.

### Computational complexity of learning ensembles of PBMs

**Fig 8:**
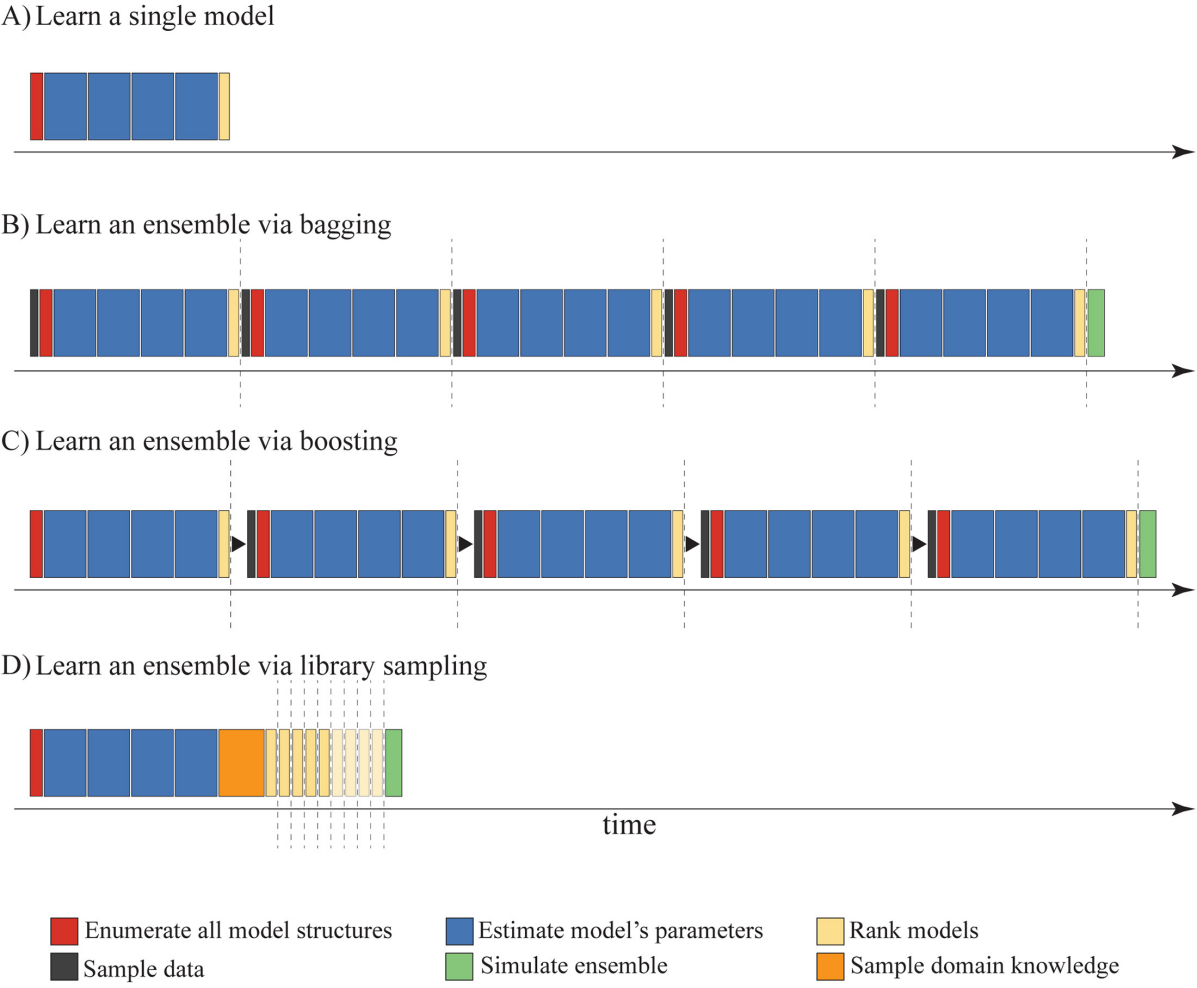
Comparison of the computational complexity of learning a single model and three methods for learning ensembles. A graphical representation of the time complexity of learning a (A) single process-based model and ensembles of process-based models with five constituents with (B) bagging, (C) boosting and (D) library sampling, using the library presented in Table 1.

Since the main focus of this study is the efficient learning of ensembles of process-based models, we first need to establish the complexity of learning a single model. Recall from Section *Process-based models*, that the algorithm for learning process-based models consists of two main sub-tasks: enumerating all possible model structures, and estimating the parameters of each of them. Fig 8A presents a diagram of relative execution times for each of the tasks through the prism of learning an example Predator-Prey model as described in Section *Process-based models*. The first task, structure enumeration (red box), uses a traversal algorithm through the space of model components, which is linear in the resulting number candidate models *N* [42]. In this example, it results in 4 candidate models. Second, for each of these candidates, a parameter estimation task is performed (blue box), the efficiency of which is related to the number of parameters each candidate model has and the number of observed time points. Note that, even though the overall complexity of the parameter estimation task is O(N), in most cases more than 99% of the computational time is spent in this phase. At the end of each modeling task, the learned models are ranked (yellow box) based on their performance on the training/validation dataset. The ranking task is performed by an insertion sort algorithm, based on the models’ performances and has a complexity of O(NlogN) (given that a sorted list of models is maintained after every model construction), where *N* is the number of candidate models. For this paper we define the complexity of one iteration of ProBMoT, i.e. obtaining one process-based model as the benchmark unit for assessing the complexity of learning different ensembles of process-based models.

For analyzing the computational complexity of the different methods for learning ensembles of process-based models, we demonstrate learning Predator-Prey ensemble models with five constituents. Fig 8B presents the time needed for learning an ensemble using the bagging method. It is essentially a repetition of the tasks needed for learning a single model for every ensemble constituent (dashed line), with the exception of two additional tasks: one for sampling the training data (gray box) at the beginning of each ensemble iteration, and one for simulating the ensemble at the end (green box). Even though Fig 8B depicts the serial implementation of bagging, this algorithm handles the processes of learning different base models completely independently. Thus, it can be parallelized to handle different tasks with different bootstrap replicates on different CPUs, which performance-wise is very useful for computationally intensive learning tasks such as process-based modeling. The complexity of learning an ensemble with boosting (Fig 8C) resembles the one of bagging. However, the boosting algorithm cannot be parallelized, as each new boosting iteration depends on the outcome of the previous one. This makes such ensembles the most inefficient to learn, which is most strongly felt when large libraries and many constituents are considered.

The last method that we investigate is learning ensembles by sampling the library of domain knowledge (Fig 8D). While the algorithm (as presented in Algorithm 1) is iterative, it can be implemented much more efficiently. Instead of sampling the space of components (i.e., sampling the domain knowledge) and running ProBMoT with each sample of the library, we can sample the generated search space and choose from the candidate models. First, all the models from the original library are generated, and their parameters are estimated accordingly. Next, we generate all the necessary library samples (orange box), and perform the task of searching and sorting models which are determined by the particular subsample of the library. By transforming the sampling problem from sampling domain knowledge to sampling the model structures from the complete search space, the number of ProBMoT runs is minimized (to 1), consequently substantially gaining computational efficiency, as compared to the other ensemble methods using ProBMoT, i.e., bagging and boosting. Earlier it was stated that selecting the constituent set for such ensembles can be performed in two ways: with and without duplicates. For the former, the execution time is correlated to the number of iterations needed (which for this example is 5). For the latter, the execution time depends on the random generator, and for this example it can take from a minimum 5 (yellow boxes) to a maximum 9 iterations (additional 4 opaque-yellow boxes). Finally, at the end, similarly to the previous approaches, an ensemble simulation task is performed.

### Summary

The results of the experiments show the following.

- The optimal design choices for the algorithm for learning library-sampling ensembles of process-based models are as follows. First, it is better to select the ensemble constituents using a separate validation dataset that has not been used to learn them (Figs 2 and 3). Second, the optimal number of constituent models is small (Fig 4). Finally, the simple average is the optimal method for combining the simulations/predictions of the ensemble constituents (Fig 5). Note that these design choices are virtually identical to the ones made for the bagging and boosting ensembles of process-based models [9].
- Library-sampling ensembles outperform the two baseline ensembles consisting of top-ten and ten randomly selected models (Fig 6). Moreover, despite the additional computational effort, removing duplicates in the set of ensemble constituents does not improve their predictive performance. Most notably, library-sampling ensembles significantly outperform single models and have comparable predictive performance to bagging (insignificantly worse average rank and slightly higher number of wins), the best performing state-of-the-art ensemble method for process-based modeling (Fig 7 and Table 2).
- The comparative analysis of the computational performances reveals that library-sampling ensembles are learned in a time comparable to the time needed to learn a single model (Figs 7 and 8). This is orders of magnitude faster when compared to its bagging and boosting counterparts, where the time needed equals the number of ensemble constituents multiplied by the time needed to learn a single model. In particular, the speed-up factor equals the number of bagged/boosted process-based models.

In sum, the library-sampling ensembles represent an important advance over the state-of-the-art methods (bagging and boosting) for learning ensembles of process-based models: They provide orders-of-magnitude improvement in computational efficiency of ensembles without impairing their predictive performance.

## Discussion

The machine learning literature provides various frameworks for explaining the performance improvement gained by using ensemble methods. While the results on the positive influence of ensemble constituents’ diversity on the performance are inconsistent [43], there is a general consensus that the bias-variance decomposition of the predictive error allows for a plausible explanation. Ensembles tend to reduce the variance component of the predictive error, while not increasing the bias component at the same time [44]. This is what happens in the case of learning ensembles of process-based models: by averaging the predictions of several models learned on the same dataset, we reduce the variance component of the predictive error. Moreover, we conjecture that ensembles of process-based models also reduce their bias. This is due to the fact that averaged process-model simulations can lead to predictions that are out of the scope of a single process-based model. Therefore, ensembles of process-based models (and models of dynamic systems in general) have the potential to extend the original space of individual models leading to a reduction of the bias component of the predictive error. Validating this hypothesis is beyond the scope of this paper, but will be considered in further work.

This paper builds upon previous work on learning ensembles for modeling dynamic systems. More specifically, it extends the scope of learning ensembles of process-based models for long-term predictive tasks, i.e., bagging and boosting [9], by introducing a novel approach for constructing efficient ensembles with satisfactory predictive performance. Second, the methods presented in this paper provide accurate predictions of the unobserved future system behavior, in contrast to Bridewell et al. [11], who build ensembles that provide accurate description of the observed system behavior.

In a broader sense, the work on library-sampling ensembles extends the state-of-the art methods for process-based modeling of population dynamics in ecology [2, 3, 14]. However, while these studies successfully for modeled the observed behavior of real-world aquatic ecosystems, the prediction of future system behavior is out of their scope. In similar context, the studies of Whigham and Recknagel [45] and Cao et al. [46] discuss the predictive performance of process-based models in a lake ecosystem. However, they assume a given model structure and by employing genetic algorithms mainly focus on the task of parameter identification of different model structures.

The novel ensemble method proposed in this paper aims at improving the generalization power of the process-based modeling approach while having reasonable computational complexity for modeling tasks with relatively large candidate model space. However, when learning such ensembles, there is a trade-off between the predictive performance of the ensemble and its understandability. Here, the gain in predictive accuracy comes at the cost of losing the understandability of the learned ensembles. Nonetheless, methods for improving the comprehensibility of ensembles can be developed, similar to the methods for integrating ensemble constituents in a single model proposed by Bridewell et al [11].

## Conclusion

In this paper, we address the task of learning ensembles of process-based models. We design, implement and evaluate a novel methodology for learning ensembles by sampling the library of domain knowledge. This methodology for learning ensembles of process-based models is the major contribution of our paper, since it improves the state-of-the-art of learning process-based models in two directions: efficiency and performance.

First, it improves the state-of-the art of learning ensembles of process-based models with a new, computationally efficient ensemble method based on library sampling. The computational efficiency of the method allows for applications to domains where a rich library of domain knowledge is available leading to a large space of candidate models. In such cases, the standard, iterative methods of bagging and boosting are not applicable due to the prohibitively high computational costs. To apply them, we need to handcraft the library of domain knowledge, omit modeling alternatives, and thus simplify the space of candidate model structures. In addition to being more efficient, the proposed method most often leads to models with better predictive performance as compared to the models obtained with the state-of-the-art ensemble methods of bagging and boosting.

Second, it improves the predictive performance of the constructed ensembles, which mainly contributes to the realm of ecological modeling. The results of the performed experimental evaluation confirm our central hypothesis that ensembles constructed via sampling the library of domain knowledge provide more accurate predictions of concentrations of species in an aquatic ecosystems than a single process-based model. This is a significant improvement of predictive performance over the state-of-the-art models of population dynamics, which, while focusing on providing an accurate explanation of the behavior of the observed system, struggle to achieve a satisfactory performance at predicting population dynamics over a long periods [4, 34]. Note that these results are consistent over experiments with data from three real-world aquatic ecosystems: Lake Bled in Slovenia, Lake Kasumigaura in Japan, and Lake Zurich in Switzerland.

While our comparative empirical study is limited to the domain of modeling population dynamics, the proposed approach to learning ensembles of process-based models is general enough to be applied to any other domain and to any other type of models of dynamic systems. To proceed with process-based modeling in other domains, one has to encode a library of domain-specific modeling knowledge. Such knowledge is available for various domains including epidemiology and modeling gene regulatory networks [47], net carbon production [48] and protein interactions [49, 50]. Some of these applications of process-based modeling show the applicability of the proposed approach to the very active fields of systems biology and bioinformatics that deal with numerous system identification problems. The efficiency and predictive performance of the library-sampling ensembles bears a promise for further applications, since combining predictive models into ensembles has been proved to work well for various computational biology problems [51–53].

We have identified several limitations of our approach that can be addressed in further work. First, note that the experiments performed in this paper are limited to modeling population dynamics in three lake ecosystems. We intend to investigate the generality of our approach and extend the scope of learning ensembles of process-based models of population dynamics to other aquatic environments, such as marine ecosystems [14] and river ecosystems [54]. Next, considering the understandability of these ensembles, we plan to follow ideas from [11] and improve our methodology by incorporating understandable structure into the resulting ensemble. Also, investigating the hypotheses about the source of the predictive performance improvement in terms of bias-variance decomposition of the predictive error, requires careful and extensive empirical analysis using the setup of Brown et al [44]. Finally, given the nature of the ensembles of process-based models learned with library sampling and bagging (i.e., sampling the feature space and sampling the data instances), and their superior performance over the individual process-based model in a predictive setting, we intend to combine these two methods. A study performed by Panov and Džeroski [27] indicates that combining two types of homogeneous ensembles, i.e. bagging and RSM, can lead to ensembles with even better predictive performance compared to each of the methods separately, while still being very efficient to construct.
